# Immunogenicity and Safety of the Recombinant Adjuvanted Herpes Zoster Vaccine in Patients with Chronic Lymphocytic Leukemia and Multiple Myeloma

**DOI:** 10.3390/vaccines12111216

**Published:** 2024-10-25

**Authors:** Panagiotis T. Diamantopoulos, Christina-Nefeli Kontandreopoulou, Christos Stafylidis, Dimitra Vlachopoulou, Stavroula Smilakou, Iraklis Patsialos, Stavroula Syriopoulou, Alexandros Gkikas, Eleftherios N. Athanasopoulos, Anastasios Vogiatzakis, Eleni Panousi, Georgios Kyriakakis, Amalia Anastasopoulou, Marina Mantzourani, Vassiliki Labropoulou

**Affiliations:** 1 Hematology Unit, First Department of Internal Medicine, National and Kapodistrian University of Athens, Laikon General Hospital, 11527 Athens, Greece; 2 Department of Microbiology, Laikon General Hospital, 11527 Athens, Greecealexandros.gikas.1994@gmail.com (A.G.);; 3 Laboratory of Biochemistry, Department of Chemistry, University of Patras, 26504 Patras, Greece; lefterhsathanasopoulos@gmail.com; 4 School of Health Sciences, Faculty of Medicine, Hematology Division, University of Patras, 26504 Patras, Greece

**Keywords:** varicella-zoster virus (VZV), chronic lymphocytic leukemia, multiple myeloma, recombinant adjuvanted herpes zoster vaccine (RZV), immunogenicity, safety

## Abstract

Background/objectives: Patients with chronic lymphocytic leukemia (CLL) and multiple myeloma (MM) are susceptible to viral infections, including varicella-zoster virus (VZV) reactivation due to both disease-related and treatment-induced immunosuppression. The recombinant adjuvanted herpes zoster vaccine (RZV) has shown high efficacy in immunocompetent adults, but immunogenicity data in CLL and MM patients are limited. This study evaluates the immunogenicity and safety of RZV in this population. Methods: Patients with CLL and MM vaccinated with RZV (administered in two doses at least one month apart) were included in the study. Pre- and post-vaccination anti-VZV IgM and IgG antibody levels were measured to assess immunogenicity, and adverse events (AEs) were captured for safety evaluation. Results: Seventy-eight patients received both vaccine doses, and 71 had post-vaccination samples. Most of the patients were IgM seronegative and IgG seropositive before vaccination. Pre-vaccination IgG levels were higher in CLL patients compared to MM patients (*p* = 0.001), while post-vaccination IgG levels significantly increased in both CLL (*p* < 0.0001) and MM (*p* < 0.0001) patients. In actively treated CLL patients, pre-vaccination IgG levels were significantly lower than in not actively treated patients (*p* = 0.002). Post-vaccination IgG levels were lower in MM patients receiving antiviral prophylaxis concurrently with the vaccination (*p* = 0.013). AEs were reported in 49.4% of patients after the first dose and 48.7% after the second dose, mostly mild (local or low-grade systemic). One case of immune thrombocytopenia was noted. Conclusions: RZV demonstrated strong immunogenicity and acceptable safety in CLL and MM patients, significantly boosting IgG levels, even in actively treated or heavily pretreated patients.

## 1. Introduction

Bacterial and viral infections represent a leading cause of morbidity and early mortality in patients with hematologic malignancies, particularly those with chronic lymphocytic leukemia (CLL) [1] and multiple myeloma (MM) [2], which are characterized by profound immunosuppression that is both disease-associated and treatment-related. CLL patients have impairments in humoral and cell-mediated immunity, which are associated with hypogammaglobulinemia, abnormalities in T-cell subsets, and defects in complement activity and neutrophil/monocyte function. These issues may be further exacerbated by the immunosuppressive effects of CLL treatments, such as chemoimmunotherapy and targeted agents [3]. For CLL patients, there are no general recommendations for antiviral prophylaxis, likely due to the heterogeneity of the patient population, while the treatment modalities and efficacy of antiviral pharmacological prophylaxis have not been evaluated in randomized controlled trials [4]. Patients with MM display impaired immune responses, mostly due to bone marrow infiltration by tumor plasma cells, along with a marked decrease in the production of normal gamma-globulin, which may be further aggravated by myeloma treatment [5]. Moreover, the use of the proteasome inhibitor bortezomib, in combination with corticosteroids or other drugs, such as the anti-CD38 monoclonal antibody daratumumab, increases the risk of viral infections [6,7]. Retrospective studies indicate that antiviral prophylaxis significantly reduces the risk of herpes zoster in these patients. The varicella-zoster virus (VZV) reactivation risk seems to be relatively low in patients with MM receiving targeted therapies, such as immunomodulatory drugs, and there is limited data on the use of antiviral prophylaxis in these patients [8,9].

Besides an increased risk of infections, previous studies have reported that patients with CLL and MM also exhibit suboptimal responses to vaccines [10,11,12]. Reactivation of latent VZV and development of herpes zoster is frequent in individuals with CLL and MM, with incidence rates of 10–15% [13] and 11–22% [14], respectively [5]. Hence, the development of prevention measures is crucial. The first herpes zoster vaccine (Zostavax vaccine, ZVL; Merck), a live-attenuated VZV vaccine, is contraindicated in immunocompromised patients due to potential virulence in those with significantly impaired immunity [5]. The vaccine’s efficacy declines with age, especially in adults 50–59 and ≥70 years [15], while it also declines within 6–8 years after vaccination [16]. In contrast, the recombinant adjuvanted herpes zoster vaccine (RZV, Shingrix^®^; HZ/su, GlaxoSmithKline Biologicals SA, Rixensart, Belgium) which has been recently approved for both immunocompetent and immunocompromised hosts, is an adjuvanted subunit vaccine consisting of a single recombinant VZV antigen, glycoprotein E (gE), and the AS01B adjuvant system [17]. RZV relies on gE alone to elicit anti-VZV immunity, with AS01B enhancing the immune response. The selection of gE as the vaccine antigen is due to its being the most abundant glycoprotein expressed by VZV-infected cells, inducing both neutralizing antibody and CD4 T-cell responses.

RZV is recommended for adults ≥50 years old based on two clinical trials that demonstrated ≥92% efficacy against herpes zoster and postherpetic neuralgia in all age groups [18]. Its efficacy has been evaluated in adults 50 years or older and showed that, with a mean follow-up of 3.2 years, vaccine efficacy was 97.2%. Similarly, in a subsequent study, with a mean follow-up of 3.7 years, vaccine efficacy was 91.3% for adults aged 70 and older [19]. A long-term follow-up study of former participants in the above trials showed that the efficacy against herpes zoster remained high for up to ten years after initial vaccination [20,21]. Data regarding hematologic patients are limited. In a phase 3, randomized, multicenter trial involving 1,846 autologous HSCT recipients, during a median follow-up of 21 months, the reduction in the incidence of herpes zoster was significant, with an incidence rate ratio of 0.32 (95% confidence interval 0.22–0.44, *p* < 0.001), corresponding to a vaccine efficacy of 68.2% [22]. Moreover, in a phase 3, randomized trial assessing the immunogenicity and safety of RZV in patients with hematologic malignancies, a post-hoc analysis revealed a vaccine efficacy of 87.2% [5,23]. Additionally, two studies with CLL patients treated with Bruton tyrosine kinase inhibitors (BTKi) demonstrated high antibody response rates to RZV [24,25]. However, collective data on the risk of herpes zoster in vaccinated CLL patients treated with novel agents are limited. The goal of the present study is to assess the immunogenicity and safety of RZV in adult patients with CLL and MM.

## 2. Methods

### 2.1. Patients

Adult patients with CLL or MM willing to be vaccinated against VZV with the new recombinant (non-live) adjuvanted shingles vaccine, according to the national vaccination program, were selected to participate in the study. Patients were treated in two university hospitals in Greece (Athens and Patras) and participated in the study after signing written informed consent. The study started in January 2024, and its scheduled duration was six months. The pre- and post- vaccination clinical and laboratory characteristics of all participants were recorded as follows: Gender at birth, age at the time of vaccination, disease duration, history of confirmed herpes zoster, prior receipt of live-attenuated zoster vaccine, and complete blood count parameters [hemoglobin (Hb) level, neutrophil, lymphocyte, monocyte, and platelet (PLT) count], gamma-globulin, and C-reactive protein (CRP) levels. Moreover, data on patients’ treatment (previous treatment lines, previous and current anticancer treatments, and antiviral medications for VZV at the time of vaccination) were also recorded. The study was approved by the Institutional Review Boards of both participating centers (Laikon General Hospital, Athens, Greece, 804/12.12.2023, and General University Hospital of Patras, 134/14.03.2024, respectively).

### 2.2. Vaccination

Patients were vaccinated with two 50 μg doses of the Shingrix^®^ vaccine, administered intramuscularly separated by at least one month according to the approved program for vaccination against VZV in immunocompromised patients. Each dose of the vaccine combines 50 μg purified gE with AS01_B_, an adjuvant system containing monophosphoryl lipid A50 μg, and QS-21 (50 μg) within liposomes [26].

### 2.3. Study Procedures

**Figure 1:**
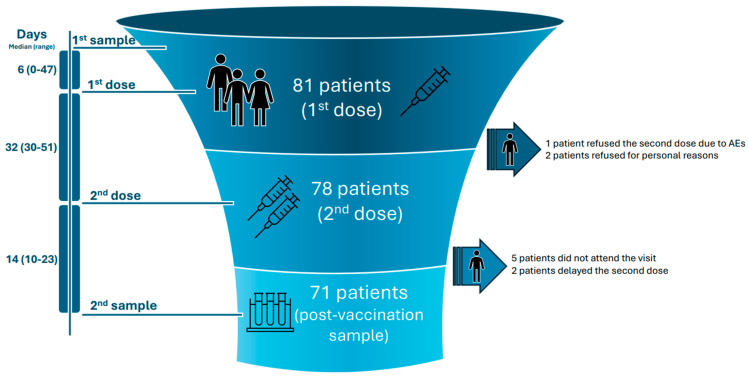
Study design diagram showing the disposition of study participants and the vaccination and sample collection timeline.

The study was designed to assess seropositivity at baseline (pre-vaccination sample) and 12–21 days after the second dose of the vaccine (post-vaccination sample). Blood samples were collected at the predefined time points. Sera were retrieved via centrifugation and stored at −80 °C. A diagram outlining the study design can be found in Figure 1.

#### 2.3.1. Immunogenicity Assessment

Sera were tested for anti-VZV IgG/IgM antibodies using the LIAISON^®^ VZV assay panel (IgG, IgM) [DiaSorin, Saluggia, Italy]. The LIAISON^®^ VZV IgG/IgM assay is a commercially available, validated indirect chemiluminescence immunoassay for the quantitative determination of specific IgG and IgM antibodies to VZV in human serum or plasma samples [27]. The sensitivity and specificity of the method are 67% and 100%, respectively. For the interpretation of the IgG assay results, a cut-off value of 150 mIU/mL was used, per the manufacturer’s instructions [28]. For the IgM assay, results were evaluated using a cut-off index value of 1, with a gray zone of +/−10% [29]. The patients were not actively followed during the post-vaccination period for breakthrough infections, but any reported herpes zoster episodes were recorded.

#### 2.3.2. Safety Follow-Up

Local or systemic adverse events (AEs) were recorded from the day of the first dose up to one month after the second dose of the vaccine. The AEs were reported or detected during the post-vaccination scheduled visit, as well as during a phone call or visit. The patients were specifically inquired about local (pain, edema, or redness at the injection site) or systemic (fever, headaches, myalgia, fatigue, malaise) AEs after receiving the first and second vaccine doses.

### 2.4. Statistical Analysis

Statistical analyses were conducted using IBM SPSS statistics, version 26 (IBM Corporation, North Castle, NY, USA). The Pearson Chi-Square test was used for associations between categorical variables, the Independent-Samples Mann-Whitney U test for testing between a categorical variable with two levels and non-normally distributed continuous variables, and the Kruskal-Wallis H test for categorical variables with more than two levels. The Wilcoxon matched-pair signed-rank test was used to compare the IgG antibody levels before and after vaccination, while the Pearson correlation was used to determine if two continuous variables were linearly related. The level of significance for all statistical tests was set at a probability value of less than 5% (*p* < 0.05, 2-sided).

## 3. Results

Eighty-one patients received at least one dose of the vaccine. Of them, 51 (63.0%) had CLL and 30 (37.0%) had MM. Seventy-eight patients eventually received both doses of the vaccine. One patient refused the second dose due to adverse events after the first dose, and the remaining two did not take the second dose for personal reasons. Eventually, 71 (87.7%) had a post-vaccination sample taken. The main reasons for not obtaining a second sample were that the patient did not attend the scheduled visit for sample collection due to personal reasons (*N* = 5), or the patient delayed the second dose and was outside the predefined time window for blood collection (*N* = 2). This data can be found in Figure 1. The baseline characteristics of the patients are shown in Table 1.

Eighteen (22.2%) patients had a history of at least one episode of herpes zoster during the course of their disease, with no rate difference between patients with CLL or MM (*p* = 0.415). Additionally, 10 (12.3%) patients had been previously vaccinated with a live-attenuated VZV vaccine, with eight of these 10 vaccinated before the onset of their neoplastic disorder. one patient with CLL had been erroneously vaccinated by his general practitioner with a live-attenuated VZV vaccine two years earlier and had subsequently suffered a severe VZV infection. No differences were noted between previously vaccinated and unvaccinated patients or between patients with and without previous herpes zoster episodes regarding their baseline characteristics. Twenty-four patients (six with CLL and 18 with MM) were under active prophylactic anti-VZV treatment with acyclovir or valacyclovir at the time of vaccination. Details on the vaccination, the timelines of the vaccine doses, and sample collections can be found in Table 2.

### 3.1. Immunogenicity Results

The pre- and post-vaccination anti-VZV IgM and IgG antibody levels are shown in Table 3. The vast majority of the patients were IgM seronegative both pre- and post-vaccination. In detail, only one patient was found seropositive for IgM before vaccination. This was an asymptomatic 75-year-old man with CLL, no history of herpes zoster, actively treated with cyclophosphamide and prednisolone, and relatively high pre-vaccination IgG antibody levels (1222 mIU/mL). The patient was IgM seronegative after vaccination. Moreover, only one patient was found seropositive for IgM after vaccination. This was an 88-year-old asymptomatic woman with MM, with no active treatment or history of herpes zoster, and relatively low pre-vaccination IgG antibody levels (396.8 mIU/mL).

**Figure 2:**
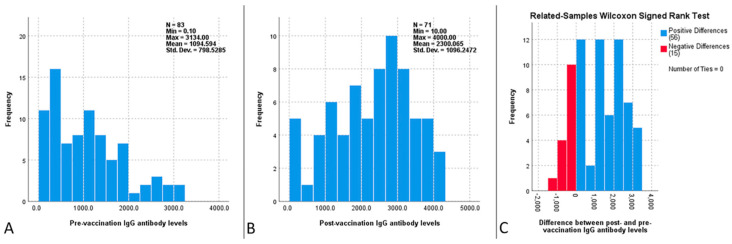
Comparison of the pre (**A**) and post (**B**) vaccination anti-VZV IgG antibody levels in the whole cohort. The difference between post- and pre-vaccination anti-VZV IgG antibody levels is also depicted (**C**).

The median pre-vaccination IgG antibody levels for the entire cohort were 937.8 mIU/mL, with CLL patients having higher pre-vaccination IgG antibody levels than MM patients (1205.5 mIU/mL vs. 466.7 mIU/mL, respectively; *p* = 0.001). Although most patients (73/79, 92.4%) had IgG antibody levels above the cut-off point of 150 mIU/mL and were considered seropositive, there was a large increase in antibody levels post-vaccination (937.8 mIU/mL vs. 2414.0 mIU/mL; *p* <0.0001), which was evident in both CLL and MM patients, as shown in Figure 2. The increase in IgG antibody levels post-vaccination was more pronounced in MM patients than in CLL patients (Table 3). No differences were found in pre- or post-vaccination IgG antibody levels between patients with and without a history of herpes zoster, or between patients previously vaccinated or not with a live-attenuated VZV vaccine. at least a fourfold increase in IgG antibody levels was observed in 29.6% of the patients, with the percentage being higher in MM than in CLL patients (57.7% vs. 15.4%, respectively, *p* < 0.001). This increase was also lower in actively treated patients than in notactively treated patients (41.7% vs. 17.1%, respectively, *p* = 0.037), but it did not differ between patients with and without a history of herpes zoster or previous vaccination with the live-attenuated VZV vaccine.

#### 3.1.1. CLL Patients

In patients with CLL, the median pre-vaccination IgG antibody levels were 1205.5 mIU/mL. The antibody levels were not associated with gender, age, or disease duration. Moreover, the antibody levels were lower in actively treated patients (*p* = 0.002), but they were not correlated with the type of active treatment. Additionally,, they were not associated with the number of previous treatment lines or previously administered anti-CD20 monoclonal antibodies, fludarabine, or BTKi. Furthermore, the levels were not associated with previous herpes zoster infection or concurrent antiviral prophylaxis with acyclovir or valacyclovir at the time of vaccination. There were no correlations with the baseline complete blood count parameters (hemoglobin level, neutrophil, lymphocyte, monocyte, and platelet count), gamma-globulin, or CRP levels. Pre-vaccination IgG seronegativity was not correlated with any of the tested baseline characteristics.

The post-vaccination IgG antibody levels were 2079.5 mIU/mL, significantly higher than the pre-vaccination levels (*p* < 0.0001). Nevertheless, there was no difference in the IgG seropositivity rate pre- and post-vaccination (96.0% vs. 97.8%). Post-vaccination IgG seronegativity was very low (2.2%) and was not correlated with any of the tested baseline characteristics of the patients. Moreover, the absolute difference between the post- and pre-vaccination antibody levels was not associated with any of the tested variables. Patients previously treated with an anti-CD20 monoclonal antibody had marginally lower IgG antibody levels than those untreated (*p* = 0.059). Nevertheless, none of the remaining baseline treatment characteristics was associated with the post-vaccination antibody levels.

Although the study was not powered to detect breakthrough infections after vaccination, there was one 82 -year-old CLL patient actively treated with venetoclax who experienced two episodes of herpes zoster post-vaccination. The patient had had three episodes before vaccination and had also been erroneously vaccinated with the live-attenuated vaccine two years earlier. His pre- and post-vaccination IgG levels did not differ from those of the whole cohort.

#### 3.1.2. MM Patients

In patients with MM, the median pre-vaccination IgG antibody levels were 466.7 mIU/mL. The antibody levels were not associated with gender, age, or disease duration. The antibody levels were similar in actively and not actively treated patients (*p* = 0.885) and were not associated with the type of active treatment or the number of previous treatment lines. Moreover, they were not associated with previously administered bortezomib, but patients previously treated with daratumumab had marginally non-significantly lower antibody levels than those not previously treated with this agent (*p* = 0.051). Finally, the pre-vaccination antibody levels were not associated with previous herpes zoster infection, but they were lower in patients receiving concurrent antiviral prophylaxis with acyclovir or valacyclovir at the time of vaccination (*p* = 0.031).

The post-vaccination IgG antibody levels were much higher than the pre-vaccination ones (2897.0 mIU/mL vs. 466.7 mIU/mL, *p* < 0.0001). Similarly to patients with CLL, the pre- and post-vaccination seropositivity rates did not differ in patients with MM (86.2% vs. 100.0%). However, it should be noted that all three seronegative patients achieved seropositivity post-vaccination. Moreover, the absolute difference between the post- and pre-vaccination antibody levels was not associated with any of the tested variables. Finally, the post-vaccination IgG antibody levels were also lower in patients receiving concurrent antiviral prophylaxis (*p* = 0.013).

### 3.2. Safety Results

AEs were reported in 40 (49.4%) patients after the first dose of the vaccine and in 38 (48.7%) after the second dose. The vast majority of the AEs were local (pain, redness, edema) (77.5% and 73.7% after each dose, respectively) or low-grade systemic ones (low-grade fever, pyrexia, headache, malaise, hypotension) (22.5% and 26.3%, respectively). Among AEs of special interest, one CLL patient presented with painful axillary lymphadenopathy ipsilateral to the vaccination site that resolved without intervention within two weeks, and another CLL patient presented 14 days after the second dose of the vaccine with grade 4 immune thrombocytopenia (ITP), which was effectively treated with intravenous immunoglobulin and high-dose dexamethasone. The patient had to be hospitalized for four days. No other hospitalizations were reported during the one-month post-vaccination period.

The emergence of AEs was not associated with most of the tested variables (gender, age, hematologic parameters, disease duration, active or previous treatments), although patients with CLL tended to report more AEs compared to patients with MM (56.9% vs. 33.3%, *p* = 0.065). patients with a history of herpes zoster reported more AEs after the second dose of the vaccine (70.5% vs. 42.6% for patients without a history of herpes zoster, *p* = 0.056). Moreover, patients with longer disease duration tended to report more AEs (*p* = 0.075), while in patients with CLL, adverse events were more common among patients with higher gamma-globulin levels (*p* = 0.017). It should be noted that the gamma-globulin levels of patients with CLL did not differ from those of patients with MM. Finally, the type of AE (local or systemic) was not associated with any of the tested variables. Still, patients with a history of herpes zoster had more systemic AEs compared to patients without such a history (55.6% vs. 12.9%, *p* = 0.025).

## 4. Discussion

Due to inherent immunodeficiency and drug-related immunosuppression, patients with CLL and MM suffer from viral and bacterial infections that pose a significant threat to their lives. Moreover, immune responses to vaccinations have been shown to be suboptimal, with associations between those responses and active or previous immunosuppressive treatments reported for several vaccines. In the present study, we analyzed the immunogenicity and safety results of RZV in a total of 81 patients (71 with post-vaccination samples) with CLL and MM.

Due to the universal infection of susceptible children in the past, when no vaccines were available against VZV, most of today’s adults have been exposed to the virus and are seropositive for IgG antibodies due to natural infection. Our study showed that the vast majority of CLL and MM patients were IgG seropositive before vaccination, although most increased their IgG levels after vaccination. This increase was statistically significant and evident in both CLL and MM patients. The percentage of patients with a fourfold increase in IgG antibody levels, though lower than that reported for immunocompetent hosts, it should be noted that there were differences in the methods used to assess immunogenicity. Although a control group was not used in the present study, the high immunogenicity rates before, but mostly after vaccination (reaching up to 98.6% in both groups), were comparable to those previously reported in phase 2 studies with the vaccine [30,31]. Cumulative data from a systematic review reports lower rates of humoral immunity in immunocompromised hosts, ranging from 33.3% to 80.4% for several immunocompromizing conditions. This study used a fourfold increase in the antibody titer to define humoral response, and according to this definition, our results are compatible with those reported in the systematic review [32]. A second meta-analysis reports only clinical outcomes [33]. The previous vaccination status (with a live-attenuated VZV vaccine) or the history of herpes zoster was not associated with pre- or post-vaccination IgG antibody levels or their increase post-vaccination. This is consistent with the fact that even patients with a history of herpes zoster may benefit from vaccination with RZV, as previously shown. Indeed, RZV elicited strong humoral responses in adults ≥50 years of age with a prior history of herpes zoster, and no safety issues were recorded [34]. Reactivation of the virus in immunocompromised hosts may be halted by the vaccine, thus preventing future herpes zoster episodes. Nevertheless, long-term follow-up to detect herpes zoster episodes was beyond the scope of the present study.

Although patients with CLL had higher pre-vaccination IgG antibody levels than patients with MM, the latter increased the IgG antibody levels in their post-vaccination samples more than the former. The fact that there were no associations of the IgG antibody levels with the baseline characteristics of the patients, not allowing for multivariate analysis, shows that there is probably an inherent difference in the antibody production ability in these two conditions, although the small patient number prevents any further analysis and speculation. Nevertheless, patients with CLL typically have poorer responses to vaccinations compared to patients with MM [35]. However, there are also studies on the immunogenicity of SARS-CoV-2 vaccines that show no differences between the two conditions [36]. In contrast to our findings, another study showed that humoral immune responses were lower in participants with non-Hodgkin B-cell lymphoma (B-NHL) or CLL compared with the entire cohort, excluding those with B-NHL and CLL stratum. The authors attributed this finding to B-cell depletion induced by therapy with anti-CD20 monoclonal antibodies [33].

In CLL patients, there was a clear difference in the pre-vaccination IgG antibody levels between patients actively and not actively treated. This is a common finding in immunogenicity studies of several vaccines, including those against SARS-CoV-2 [37]. This result is contradictory to that of a study on 106 CLL patients (50 under a BTKi and 56 treatment-naïve) vaccinated with RZV, where a clear difference in the immunogenicity of the vaccine was noted between the two groups [38]. Nevertheless, in another smaller study, BTKi did not have any effect on the immunogenicity of the vaccine [25], while in a third one, although BTKi were associated with reduced cellular immune responses in the univariate analysis, a multivariate analysis was not conducted [39]. It should also be noted that active treatment did not affect immunogenicity in patients with MM. In the above-referenced study by Dagnew et al. [37] with patients suffering from several hematologic conditions, patients vaccinated after completing their immunosuppressive treatment had higher rates of humoral response than those vaccinated during their course of immunosuppressive treatment. This result was not highlighted in the present study, maybe due to the small patient number in each disease category.

In patients with MM, although immunogenicity was not associated with the baseline and treatment characteristics, there was a lower pre- and post-vaccination IgG antibody level in patients actively treated with anti-VZV agents during their vaccination. This interesting finding is hard to interpret since the vaccine is not contraindicated in patients under antiviral prophylaxis while there should not be any implication of the antiviral treatment in the ability of the immune system to recognize antigens and build an immune response. Perhaps prophylactic treatment with anti-VZV agents prevents low-level viremia/subclinical reactivation, further boosting humoral and cellular immunity [40]. Nevertheless, this result was not evident in patients with CLL. In any case, until more data is available on the efficacy of the vaccine in these populations, patients in need of anti-VZV prophylaxis should continue prophylactic treatment even if they are vaccinated with RZV.

Regarding the safety of the vaccine, most reported AEs were local or low-grade systemic ones, with the exception of a presumably vaccine-associated case of ITP. Most vaccines are not linked to an increased risk of ITP except for MMR in children [41] and possibly COVID-19 vaccines in middle-aged and elderly individuals, especially those with chronic inflammatory conditions [42]. A flare of an underlying rheumatologic disease or triggering of an immune-mediated condition is a concern with adjuvanted vaccines. Nevertheless, exacerbations of the underlying disease were uncommon in rheumatologic patients vaccinated with RZV. Additionally, potential autoimmune phenomena after the administration of RZV in patients with hematologic malignancies have been reported in only 1.1% of vaccinated patients [43,44]. The fact that patients with CLL and higher gamma-globulin levels reported more AEs than those with lower gamma-globulin levels could be explained by the idea that less immunosuppression may lead to enhanced local or systemic reactions, but this remains speculative. Nevertheless, it should be mentioned that data from a meta-analysis of several studies showed that AEs did not differ significantly between immunocompetent and immunocompromised adults vaccinated with RZV, though no data on gamma-globulin levels were available [45]. Finally, the finding that patients with a history of herpes zoster had higher rates of systemic AEs than those without such a history is interesting but difficult to interpret in light of the present study’s findings.

The safety results of RZV were consistent across several studies [46,47,48,49]. In the ZOE-50 and ZOE-70 trials, injection site pain emerged as the most frequent local AE, reported in 68–70% of adults aged ≥50, with similar results observed in immunocompromised patients (83–87%). Myalgia and fatigue were the most common systemic AEs across both groups, with a notably higher incidence in immunocompromised patients (74% for myalgia and 49.6% for fatigue vs. 32% in older patients). Grade 3 AEs were less common in older patients (≤4% vs. 13–14% for immunocompromised patients). SAEs did not differ between healthy and immunocompromised patients [50,51]. In the remaining studies, no significant differences in AE rates were observed between immunocompromised patients and healthy individuals, and the AE duration was short.

The results of the present study provide valuable insights into the immunogenicity and safety of RZV in patients with CLL and MM. Nevertheless, there are some limitations to the study. First, the lack of a control group prevents comparisons of immunogenicity data between CLL/MM patients and healthy individuals. We have attempted to compare our results with the immunogenicity results of phase 2 studies of the vaccine. Second, the relatively small sample size which may not be representative of the variability of responses in this population. A larger cohort would increase the statistical strength and robustness of the results. Moreover, a sample size estimation was not performed during the design of the study, as it was not a multicenter study across a broad number of institutions but was conducted only in two university hospitals. This limited the number of patients eligible to participate. For the same reasons, there were some differences in the baseline characteristics between the two groups of patients; most of these differences were inherent to the disease and its treatment. Finally, While the study focuses on short-term AEs, it lacks long-term follow-up to assess the duration of immune responses and potential delayed AEs.

## 5. Conclusions

The present study shows that patients with CLL and MM increase their levels of anti-VZV IgG antibodies when vaccinated with RZV, even if they are actively treated or heavily pretreated. The immunogenicity of the vaccine is strong and not correlated with most of the baseline characteristics of the patients. Nevertheless, these results, taken together with the available data on the vaccine’s ability to prevent herpes zoster and postherpetic neuralgia in immunocompromised individuals, cannot serve as a basis for omitting prophylactic anti-VZV treatment with antivirals in these patients. Results from larger studies focusing on breakthrough herpes zoster episodes may provide valuable insights in this context.

## Figures and Tables

**Table 1:** Baseline patient and disease characteristics and treatment data are presented in this table for the whole cohort, but also separately for CLL and MM patients.

Characteristic	All Patients	CLL	MM	*p* *
Number of patients, N (%)	81 (100.0)	51 (63.0)	30 (37.0)	NA
Gender (male/female)	1.19 (44/37)	1.32 (29/22)	1.00 (15/15)	0.658
Age (years), median (range)	74 (44–88)	73 (44–86)	74 (50–88)	0.935
Time since diagnosis (years), median (range)	5 (1–31)	8.0 (1–31)	3.5 (1–19)	<0.001
Previously treated, N (%)	NA	30 (58.8)	25 (83.3)	0.001
Previous treatment lines, N (%)	NA			0.058
0		21 (41.2)	5 (16.7)	
1		13 (25.5)	7 (23.3)	
2		12 (23.5)	11 (36.7)	
3		5 (9.8)	5 (16.7)	
>3		0 (0.0)	2 (6.7)	
Previous anti-CD20, N (%)	NA	20 (39.2)	NA	NA
Previous fludarabine, N (%)	NA	3 (5.9)	NA	NA
Previous BTKi, N (%)	NA	6 (11.8)	NA	NA
Previous BCL2i, N (%)	NA	0 (0.0)	NA	NA
Previous proteasome inhibitor, N (%)	NA	NA	24 (80.0)	NA
Previous daratumumab, N (%)	NA	NA	15 (50.0)	NA
Actively treated	42 (51.9)	19 (37.3)	23 (76.7)	0.001
Type of active treatment, N (%)	NA			NA
Venetoclax		8 (15.7)		
Acalabrutinib		3 (5.9)		
Ibrutinib		6 (11.8)		
Rituximab		1 (2.0)		
COP		1 (2.0)		
Ant-CD38/KD			4 (13.3)	
Lenalidomide			8 (26.7)	
VRD/KPD			2 (6.7)	
Anti-CD38-lenalidomide			3 (10.0)	
Selinexor-lenalidomide			1 (3.3)	
Anti-CD38			3 (10.0)	
Belantamab mafodotin			1 (3.3)	
Anti-CD38-ixazomib			1 (3.3)	
History of herpes zoster, N (%)	18 (22.2)	13 (25.5)	5 (16.7)	0.415
Previous herpes zoster episodes				0.867
0	63 (77.8)	38 (74.5)	25 (83.3)	
1	14 (17.3)	11 (21.6)	3 (10.0)	
2	3 (3.7)	1 (2.0)	2 (6.7)	
3	1 (1.2)	1 (2.0)	0 (0.0)	
Time since last herpes zoster episode (months), median (range)	113.3 (3.0–410.0)	171.8 (3.0–410.0)	58.0 (4.0–338.0)	0.054
Previously vaccinated with live VZV vaccine, N (%)	10 (11.9)	7 (13.5)	3 (9.4)	0.735
Time since live VZV vaccination, N (%)	51.8 (42.0–98.0)	51.1 (42.0–98.0)	57.2 (51.0–74.0)	0.517
Previous anti-VZV agent, N (%)	33 (40.7)	11 (21.6)	22 (73.3)	<0.001
Prophylaxis, N (%)	32 (97.0)	10 (90.9)	22 (100.0)	
Treatment, N (%)	1 (3.0)	1 (10.1)	0 (0.0)	
Active anti-VZV agent, N (%)	24 (40.7)	6 (11.8)	18 (60.0)	<0.001
Time on anti-VZV agent at 1st vaccine dose (months), median (range)	27.7 (1.1–230.4)	25.3 (1.1–109.4)	28.0 (2.5–230.4)	0.513
Hemoglobin level (g/dL), median (range)	13.1 (9.6–16.5)	13.5 (9.6–16.5)	12.2 (10.3–15.2)	0.085
WBC count (×10^9^/L), median (range)	7.1 (1.3–178.2)	13.2 (1.3–178.2)	5.9 (2.7–13.1)	<0.001
Neutrophil count (×10^9^/L), median (range)	3.4 (0.5–10.5)	3.6 (0.5–10.5)	3.1 (1.0–8.9)	0.374
Lymphocyte count (×10^9^/L), median (range)	2.3 (0.3–173.4)	6.9 (0.5–173.4)	1.5 (0.3–4.5)	<0.001
Monocyte count (×10^9^/L), median (range)	0.6 (0.2–6.7)	0.7 (0.2–6.7)	0.5 (0.2–1.2)	0.02
Platelet count (×10^9^/L), median (range)	170 (84–442)	177 (84–372)	166 (86–442)	0.637
gamma-globulin level (g/L), median (range)	5.2 (1.4–23.7)	6.3 (1.5–23.7)	3.1 (1.4–14.3)	0.081
CRP level (mg/dL), median (range)	0.9 (0.1–18.6)	0.8 (0.1–18.6)	1.2 (0.2–8.5)	0.734

CLL, chronic lymphocytic leukemia; MM, multiple myeloma; NA, not applicable; BTKi, Bruton tyrosine kinase inhibitor; BCL2i, B-cell lymphoma 2 inhibitor; VZV, varicella-zoster virus; WBC, white blood cell; CRP, C-reactive protein. Treatment regimens: COP, cyclophosphamide, vincristine, prednisone; VRD, bortezomib, lenalidomide, dexamethasone; KPD, carfilzomib, pomalidomide, dexamethasone. * 2-sided p referring to comparisons between CLL and MM patients.

**Table 2:** Vaccination and safety data.

Characteristic	All Patients	CLL	MM	*p* *
First sample collection, N (%)	81 (100.0)	51 (63.0)	30 (37.0)	NA
Administration of first vaccine dose, N (%)	81 (100.0)	51 (63.0)	30 (37.0)	NA
Time from first sample collection to first vaccine dose (days), median (range)	6 (0–47)	5 (0–46)	9.5 (1–47)	0.103
Adverse events after first dose, N (%)	40 (49.4)	29 (56.9)	10 (33.3)	0.065
Local	31 (38.3)	21 (41.2)	9 (30.0)	
Systematic	9 (11.1)	8 (15.7)	1 (3.3)	
Administration of second vaccine dose, N (%)	78 (96.3)	50 (98.0)	28 (93.3)	NA
Time between the two vaccine doses (days), median (range)	32 (30–51)	32 (30–51)	31 (30–45)	0.991
Adverse events after second dose, N (%)	38 (48.7)	27 (54.0)	11 (36.7)	0.212
Local	28 (35.9)	18 (35.3)	10 (33.3)	
Systematic	10 (12.8)	9 (17.6)	1 (3.3)	
Second sample collection, N (%)	71 (87.7)	45 (88.2)	26 (86.7)	NA
Time from second vaccine dose to second sample collection (days), median (range)	14 (10–23)	13 (10–22)	15 (10–23)	0.067

CLL, chronic lymphocytic leukemia; MM, multiple myeloma. * 2-sided p referring to comparisons between CLL and MM patients.

**Table 3:** Anti-VZV IgM and IgG antibody levels in CLL and MM patients included in the study.

Antibody Levels (mIU/mL), Median (95% CI)	All Patients	CLL	MM	2-Sided *p* *
Pre-vaccination				
IgG levels	937.8 (0.1–3134.0)	1205.5 (0.1–3134.0)	466.7 (44.7–1587.0)	0.001
IGM levels	0.1 (0.0–1.0)	0.13 (0.0–1.0)	0.14 (0.0–1.0)	0.486
Post-vaccination				
IgG levels	2414.0 (10.0–4000.0)	2079.5 (185.0–4000.0)	2897.0 (10.0–3957.0)	0.035
IGM levels	0.2 (0.0–1.0)	0.2 (0.1–0.8)	0.25 (0.1–1.6)	0.383
**Seropositivity, N (%)**	**All Patients**	**CLL**	**MM**	2-Sided *p* *
Pre-vaccination				
IgG	73 (92.4)	48/50 (96.0)	25/29 (86.2)	0.185
IgM	1 (1.3)	1/50 (2.0)	0/29 (0.0)	1.000
Post-vaccination				
IgG	70 (98.6)	44/45 (97.8)	26/26 (100.0)	1.000
IgM	1 (1.4)	0/45 (0.0)	1/25 (4.0)	0.366

CI, confidence intervals; CLL, chronic lymphocytic leukemia; MM, multiple myeloma. * referring to comparisons between CLL and MM patients.

## Data Availability

The raw data supporting the conclusions of this article will be made available by the authors on request.
